# Effect of Judo Athletes’ Psychological Function on Sports Coping Skills: Moderated Mediating Effect of Tension

**DOI:** 10.3390/ijerph19116634

**Published:** 2022-05-29

**Authors:** Hye-Young Kim, Eun-Jung Chung, Sang-Woo Kim, Byoung-Hee Lee

**Affiliations:** 1 Graduate School of Physical Therapy, Sahmyook University, Seoul 01795, Korea; young9618@hanmail.net; 2 Department of Physical Therapy, Andong Science College, Andong 36616, Korea; qkskskzl@nate.com; 3 Virtual Rehabilitation Lab, Sahmyook University, Seoul 01795, Korea; adonis2023@naver.com; 4 Department of Physical Therapy, Sahmyook University, Seoul 01795, Korea

**Keywords:** self-management, sports athletes, psychological factors, sport coping

## Abstract

This study aimed to investigate the effects of Judo athletes’ psychological function on sports coping skills through self-management: the moderated mediating effect of tension. A total of 124 participants (66 males and 58 females) were included, comprising high school students, college students, and judo team players (age 16 to 30, 20.51 ± 3.17) in the Republic of Korea. The psychological function was measured using the Profile of Mood Test, Athletes’ Self-Management Questionnaire, and Athletic Coping Skills Inventory-28. The results of the analysis of the moderating effect of the athlete’s self-management behavior showed that tension had a moderating effect on the relationship between the athlete’s self-management behavior and sports coping skills. The mediating effect analysis revealed a mediating effect of self-management behavior on the relationship between player vitality and sports coping skills. It was also confirmed that tension had a moderating effect on athletes’ self-management behavior and sports coping skills. Therefore, it was confirmed that the higher the self-management, the more moderated the mediating effect on sports coping skills. In conclusion, it was confirmed that psychological function affects sports coping skills, and thereby, the mediating effect of the athlete’s self-management behavior is regulated by tension. In future research, it will be necessary to study the sports coping ability and performance of judo athletes according to tension control.

## 1. Introduction

Athletes not only lose their game coping skills due to an athletic injury but also put in time and money to return to exercise after the injury occurs [1]. The first step in preventing such an injury is to analyze the cause of the injury. In analyzing these factors, they are classified into internal and external risk factors [2]. Internal risk factors refer to the biological, psychological, and social characteristics of an individual that result in an injury, and external risk factors refer to the external influences on athletes, such as physical factors [3,4]. Psychological factors are internal risk factors for overuse injuries and stress-induced acute injuries. Although previous studies were limited to examining acute injuries, studies on the relationship between psychological factors and overuse (chronic) injuries have recently increased [5]. Currently, an intervention technique that utilizes appropriate physical and psychological factors is needed to prevent injury. Injury prevention can be seen as the foundation for good performance by providing athletes with the best conditions [6,7].

In sports psychology, athletes need to take good care of themselves to maintain their best condition. Self-management refers to a process in which athletes use successful coping skills to lead change by using holistic strategies to manage themselves in terms of their body and mind, training, and handover in daily life and training situations. In addition, by making an effort to change a certain behavior, he/she corrects the environmental conditions and adjusts and organizes the behavioral outcomes [8]. It can be said that self-management is necessary for judo athletes who are frequently injured, which can lead to major injuries. The higher the self-management, the higher the sports coping skills. The self-management of athletes is closely related to and affects self-confidence [8]. When self-management is good, it can be said that “individuals experience a positive mood and increased confidence in coping skills.” Self-management encompasses personal, internal, external, and environmental factors and is a psychobehavioral strategy, which means that individuals become thorough in everything, from physical and mental aspects to training and their personal life, to achieve their goals [9].

The higher the positive mental state of the athlete, which is a psychological factor as well as a physical factor, the higher the rate of returning to sports after an exercise injury [10]. It has been reported that the higher the positive emotions compared to negative emotions, the more positive the exercise management skills strategy. In addition to these positive psychological factors, it has been reported that athletes who are better at self-management have better motor managing skills [11]. According to the results of previous studies [12], various strategies such as positive thinking, effective training, and thorough psychological preparation for matches are directly linked to successful performance, and positive emotions are also important to athletes. Additionally, research on psychological counseling, which is a mediator of sports psychology and an internal risk factor, has been actively conducted. However, studies on the moderating or mediating effect between psychological function and sports psychological factors are rare [13], and there are no papers on which psychological factors affect sports coping ability. Therefore, in this study, the moderating effect of psychological factors on the sports coping ability of judo athletes was verified.

## 2. Materials and Methods

### 2.1. Participants and Experimental Procedure

A total of 124 Korean judo athletes were recruited from high school, university, and business judo teams in the Republic of Korea. This study used a self-written questionnaire survey. To determine the influence of judo athletes’ psychological functions on sports coping skills, the related literature was reviewed, and for empirical research, a survey was conducted through a self-written questionnaire to measure the psychological functions of judo athletes in high school, university, and business teams. Before recruiting participants for this study, we performed a power analysis using G*Power Version 3.1.9.7 (Franz Faul, University of Kiel, Kiel, Germany, 2020). The overall effect size index for Critical z was 0.5234942, and actual power was 0.7015140 with an odds ratio of 1.3, Pr (Y = 1|X = 1) H0 of 0.2, α err prob of 0.3003152 and power (1 − *β* err prob) of 0.6996848. Since the estimated target sample size was 101, we recruited 124 participants for this study.

The participants of this study were 124 male (*n* = 68) and female (*n* = 58) judo athletes from high school, university, and business teams (age 16 to 30, 20.51 ± 3.17). A mood status test (Profile of Mood Test; POMS) for psychological measurement, Athletes’ Self-Management Behavior Test (ASMQ), and Exercise Coping Skills Test (Athletic Coping Skills Inventory-28; ACSI-28) were conducted. A total of three questionnaires (POMS, ASMQ, and ACSI-28) were administered and analyzed to determine the moderated mediating effect of self-management on the influence of judo athletes’ psychological functions on sports coping skills. The general characteristics of the study participants are presented in Table 1.

The present study was approved by the Sahmyook University Institutional Review Board (2-1040781-AB-N-01-2017065HR), and it was registered (KCT0005885) in the Clinical Research Information Service of the Republic of Korea. The objective of the study and its requirements were explained to the subjects, and all participants provided consent. This was in accordance with the ethical principles of the Declaration of Helsinki.

### 2.2. Outcome Measures

Psychological function was measured using the POMS, ASMQ, and ACSI-28.

The POMS was used to measure an individual’s temporary mood state [14,15] (Table 2). Six factors, namely tension, depression, anger, vitality, fatigue, and confusion, were measured, and a total of 65 questions, composed of adjectives expressing mood, were answered on a 5-point Likert scale (0: not at all; 4: very much). Questions 22 and 54 were calculated as inverse values, and all values of tension, depression, anger, fatigue, and confusion were added, and the value of vitality was subtracted in the total mood disorder test score. Of the 65 questions, the remaining 7 questions were not answered, and only 58 questions were answered. The higher the score, the higher the negative mood. The six subvariables of the mood status questionnaire were as follows: First, tension is a measure of a mood that is externally uncomfortable or restless, psychologically agitated, or anxious due to muscle tension. Second, depression is a measure of feeling depressed, resigned, unhappy, or personally discontent. Third, anger is a measure of a feeling of being grumpy or distorted because of anger and resentment toward others. Fourth, activity is a measure of a feeling of energetic, joyful, pleasant, and vigorous mood. Fifth, fatigue is a measure of feeling tired, exhausted, and unmotivated. Sixth, confusion is a measure of feeling uncertain or confused due to distraction [16].

The ASMQ was used to evaluate changes in self-management behavior [17,18] (Table 3). This questionnaire was developed to measure six areas in athletes (physical conditioning, training management, mental readiness, daily living management, interpersonal relationship, and personal routine), composed of a total of 35 positive questions. The questions were answered on a 5-point scale (1 point, no; 5 points, yes). The six subvariables of the athlete self-management behavior test sheet are as follows: First, physical conditioning is a self-strategy related to behaviors necessary for body management, such as injury prevention, healthy eating, nutritional supplementation, and sufficient sleep. Second, training management is a self-management strategy for athletes to improve their physical strength and skills through regular training and insufficient personal practice during normal training. Third, mental readiness is a self-management strategy that includes inspiring self-confidence and having positive thoughts to overcome negative emotions such as anxiety and demonstrating successful performance in highly competitive sports. Fourth, daily living management is a self-management strategy related to correct behavior in life, such as regular life, polite behavior, and initiative behavior. Fifth, interpersonal relationship management is a self-management strategy to maintain smooth relationships with parents, friends, seniors and juniors, and leaders, which are important to players themselves. Sixth, personal routine is a self-strategy that thoroughly adheres to the individual’s unique behaviors, such as reducing tension, practicing according to their training schedule, and learning theories.

The ACSI-28 was used to evaluate sports coping skills [19,20] (Table 4). The ACSI-28 is composed of seven subvariables, including coping with adversity, peaking under pressure, goal setting/mental preparation, concentration, freedom of worry, confidence and achievement motivation, and coachability, and consists of a total of 28 questions with four items for each subscale. The participants were asked to respond to the questions using a 4-point Likert scale (1 point, not at all; 4 points, always).

### 2.3. Statistical Analysis

Statistical analysis was performed using SPSS ver. 24.0 (SPSS Inc., Chicago, IL, USA). First, frequency analysis, descriptive statistics, and correlation analyses were performed to examine the characteristics of the variables. Second, the moderating effect, mediating effect, and regulated mediating effect were sequentially verified for the analysis of the modulated mediating effect. The moderating effect was verified using PROCESS macro model 1, the mediating effect using PROCESS macro model 4, and the modulated mediating effect using PROCESS macro model 14 [16,17]. The bootstrap confidence interval was used to verify that the indirect effect of the mediation model was statistically significant. For all tests, the level of statistical significance was set at *p* < 0.05.

## 3. Results

### 3.1. Analysis of the Moderating Effect

The effect of tension control on athletes’ self-management on sports coping skills was verified (Table 5). A statistically significant effect of athletes’ self-management on sports coping skills was observed (B = 0.370, *t* = 8.377, *p* < 0.001), and when tension acted as a moderating variable, self-management and sports coping skills were found. A moderating effect was noted (B = −0.028, *t* = −3.609, *p* < 0.001).

The degree to which the input variables explain the dependent variable can be considered to have an explanatory power of 5.9% based on R^2^, and the model fit was also significant (*F* = 33.764, *p* < 0.001). Therefore, it was found that tension had a moderating effect on the relationship between athletes’ self-management and sports coping skills.

### 3.2. Analysis of the Mediated Effect

Data were analyzed using SPSS Macro PROCESS (model 4) developed by Hayes to verify the mediating effect of athletes’ vitality on sports coping skills through self-management [21,22]. The mediating effect of athletes’ vitality on sports coping skills through self-management was verified. The results showed that the effect of vitality as an independent variable on self-management as a parameter (path a) was statistically significant (B = 0.888, *t* = 3.291, *p* < 0.01), and in the state of controlling vitality, the effect of self-management as a parameter on the dependent variable, sports coping skills (b), was also statistically significant (B = 0.354, *t* = 7.843, *p* < 0.001). The effect of vitality on sports coping skills in a controlled self-management state (c’) was also statistically significant (B = 0.520, *t* = 3.709, *p* < 0.001).

In addition, the total effect of the independent variable, vitality, on the dependent variable, sports coping skills, without considering the parameter self-management was also significant (B = 0.833, *t* = 5.074, *p* < 0.001). It was confirmed that the total effect (c) was greater than that of the direct effect (c’). Therefore, self-management was statistically significant in the relationship between the athletes’ vitality and sports coping skills, and the direct effects of the athletes’ vitality and sports coping skills also showed statistical significance. It was verified that self-management plays a role as a mediator in athletes’ vitality and sports coping skills.

Based on these results, the significance of the mediating effect was verified, and the lower limit confidence interval and upper limit confidence interval values did not have a value of 0 in the 95% confidence range. Therefore, the mediating effect of self-management was statistically significant. Therefore, the mediating effect of athletes’ vitality on sports coping skills through self-management was statistically significant, and the direct effect of the athletes’ vitality on sports coping skills was also significant, supporting the partial mediation model. The results of the mediating effects are presented in Table 6. The significance of the indirect effect was confirmed to be significant by Z = 3.013 (*p* < 0.01) through the Sobel Z-test.

### 3.3. Verification of the Moderated Mediation

The moderated mediating effect was verified on the influence of athletes’ vitality on sports coping skills through self-management. To verify the mediating effect of self-management, which differs by tension, on the athletes’ vitality on sports coping skills, each variable was input according to the statistical model, and the following result values were derived (as presented in Table 7).

First, self-management was set as a dependent variable, and vitality was used as an independent variable for the analysis. The results showed that the effect of self-management on resilience had a statistically significant effect through the regression coefficient (B = 0.887, *p* < 0.01). Based on R, the degree to which the input independent variable can explain the dependent variable had an explanatory power of 8.2%, and the model fit was also confirmed to be significant (*F* = 10.831, *p* < 0.01).

Second, sports coping skills as a dependent variable, vitality as an independent variable, self-management as a parameter, and tension as a control variable were analyzed. The regression coefficient of vitality (B), the independent variable, was 0.526 (*p* < 0.001); the regression coefficient of self-management (B), the parameter, was 0.636 (*p* < 0.001); and the regression coefficient of tension (B) was 2.543 (*p* < 0.05). The variables had a significant effect on the dependent variable of sports coping skills. In addition, the regression coefficient (B) of the interaction term between self-management and tension was −0.023 (*p* < 0.01), which was found to have a negative (−) influence on the dependent variable. The degree to which the input independent variable could explain the dependent variable had an explanatory power of 51.6%, and the model fit was also confirmed to be significant (*F* = 31.670, *p* < 0.001). These results indicate that athletes’ vitality has a significant relationship with sports coping skills through self-management, and the mediating effect of self-management is controlled by the athlete’s tension.

In the controlled parameter model of this study, the focus was on estimating the indirect effect of the interaction term of the independent variable on the dependent variable through the parameter [22]. Therefore, the magnitude of the indirect effect affecting self-management using the condition value of tension and the results thereof is shown in Table 8. The results of the analysis showed that the direct effect of vitality on sports coping skills was 0.526 (*p* < 0.001), and even in the verification of the significance level of bootstrapping through 95% CI, it was found to be statistically significant because 0 in the confidence interval was not included. Next, to examine the conditional indirect effects of vitality → self-management → sports coping skills according to the level of tension, the level of tension was divided into three groups: average and ±1 SD. As a result, M − 1 SD = 0.395, M = 0.283, and M + 1 SD = 0.171 were found to be significant in all three groups. In addition, this was confirmed to be statistically significant as it did not include 0 in the confidence interval, even in the verification of the significance level through 95% CI.

It can be said that the most important part of the inference test of the adjusted mediated analysis lies in the index of moderated mediation. The overall size (moderated mediation index) of the indirect effect of X (vigor) → M (self-management) → Y (sports coping skills) was −0.020, which is a conditional indirect effect regulated by V (tension); the 95% bootstrap confidence interval was [−0.040 to −0.006]; and since 0 was not included in the confidence interval, it can be interpreted that the conditional indirect effect of this study was significant (Table 9).

## 4. Discussion

This study was conducted to verify the moderated mediating effect of self-management on the effects of judo athletes’ psychological functions on sports coping skills. In this study, 124 judo athletes participated, composed of high school students, college students, and business team athletes. As psychological tests, the POMS, ASMQ, and ACSI-28 were used. This study aimed to investigate the moderated mediating effect of self-management on the effects of judo athletes’ psychological functions on sports coping skills.

First, we investigated whether psychological function affects sports coping skills. Self-management behavior, vigor of mood state, tension, and intrinsic behavior management of self-management behavior influenced sports coping skills. Athletes’ self-management behavior, vigor, and tension of psychological tests affected sports coping skills, but sports competition anxiety did not affect sports coping skills. These results are consistent with the results of Joung [23]. It was possible to infer that self-management affects sports coping skills, and the better the self-management of athletes, the better their sports coping ability. The conceptual definition of self-management is said to be a series of processes of mentally preparing for and overcoming not only everyday life but also sports situations through self-control and self-regulation in one’s life [8,24]. Judo is considered training rather than exercise, and the purpose of training is not only to cultivate skills and physical strength but also to cultivate a correct personality and overcome difficulties. For judo athletes, self-management is an element that is lost in the process of training. The more self-managed players are, the higher their sports coping skills.

Second, by examining the moderating and mediating effects of psychological function on sports coping skills, it was found that tension has a moderating effect on the effect of self-management on sports coping skills. Regarding the influence of psychological function on sports coping skills, a controlled mediating effect was also found in which the mediating effect of self-management was changed to tension. The results showed that the higher the tension, the higher the self-management, and the higher the sports coping skills. The degree of tension was high in high-risk athletes, such as ski, ice hockey, and American football players, but low in non-contact sports participants [25]. Judo is also a martial arts sport, and it can be said to have high tension as a high-risk exercise. In this study, intensive self-management was considered a factor of high tension, and the better the self-management, the more directly or indirectly performance was affected. In a sports situation, self-management is the process of preparing and overcoming oneself through moderation, which means that a player who is better at self-management can demonstrate a successful performance. This can increase exercise performance and improve self-management, supporting the results of this study [26].

As a result of analyzing the mediating effect of self-management on the relationship between athletes’ vigor and sports coping skills, self-management was significant in the relationship between athletes’ vigor and sports coping skills, and the direct effects of athletes’ vigor and sports coping skills were also significant. This study shows that self-management plays a role in mediating athletes’ vigor and sports coping skills. This means that if a player with positive emotional vigor is good at self-management, he or she can cope with sports well. In other words, it was found in the same context as previous studies that improvement in athletes’ psychological variables would improve performance [27]. Athletes with high self-management skills have a high level of satisfaction with exercise and have high sports coping skills in training or competition situations [9]. In a study by Lee et al. [28], examining the self-management and anxiety of competition in Taekwondo breaking athletes, it was found that the higher the self-management of athletes is, the lower the anxiety and the higher the degree of exercise immersion, which are partially consistent with the results of this study.

Athletes’ vigor has a significant relationship with sports coping skills through self-management, and the mediating effect of such self-management is controlled by the athlete’s tension. The mediating effect of self-management was significant in the influence of positive emotions of judo athletes on sports coping skills. Regarding the effect of self-management on sports coping skills, it was also confirmed that tension has a modulating effect. Lee [29] reported direct and indirect effects on performance strategies through the positive and negative emotions of boxers. The results of this study were partially consistent with those in this study in that positive emotions directly and indirectly affected performance strategies. A previous study showed that players who were assessed as challenging in a stressful game situation promoted positive emotions, whereas those who were evaluated as threatening, through aspects such as a loss of value and loss in the game, had increased negative emotions [30,31]. It is thought that in the high-risk judo athletes in this study, positive emotional vigor directly and indirectly influenced sports coping skills.

A limitation of this study was that the questionnaire was not conducted immediately before or after a game. Although there was a limitation in that the number of participants was small, in this study, the moderated mediating effect of self-management could be confirmed through the effect of the physical and psychological functions of judo athletes on sports coping skills. This study aimed to provide data for the prevention of injuries and improvement of athletic performance in athletes.

This study was conducted to verify the controlled mediating effect of self-management on the psychological function of judo athletes on sports coping skills. According to the analysis results regarding the moderating effect of an athlete’s self-management behavior, tension had a modulating effect on the relationship between athletes’ self-management behavior and sports coping skills. As a result of the mediating effect analysis, a mediating effect of self-management behavior on the relationship between athletes’ vigor and sports coping skills was observed. Regarding the effect of self-management behavior on sports coping skills, it was also confirmed that tension has a moderating effect.

## 5. Conclusions

This study was conducted to verify the controlled mediating effect of self-management on the psychological function of judo athletes on sports coping skills. According to the analysis results of the moderating effect of athletes’ self-management behavior, tension had a modulating effect on the relationship between athletes’ self-management behavior and sports coping skills. The mediating effect analysis showed that self-management behavior had a mediating effect on the relationship between athletes’ vigor and sports coping skills. Regarding the effect of self-management behavior on sports coping skills, it was also confirmed that tension has a moderating effect. Therefore, the higher the self-management, the more moderated the mediating effect on sports coping skills. In conclusion, it was confirmed that psychological function affects sports coping skills, and thereby, the mediating effect of athletes’ self-management is controlled by tension. Therefore, it is thought that a study on sports coping ability and competition performance according to the tension control of judo athletes is necessary in a future paper.

## Figures and Tables

**Table 1:** General characteristics of participants (*n* = 124).

Characteristics	Mean ± SD
Sex (male/female)	66/58
Ages (years)	20.51 ± 3.17
Height (cm)	169.57 ± 8.32
Weight (kg)	76.86 ± 21.70

**Table 2:** Profile of Mood State (POMS).

Subscale	Item Number	
Tension	2, 10, 16, 20, 22 *, 26, 27, 34, 41	9 items
Depression	5, 9, 14, 18, 21, 23, 32, 35, 36, 44, 45, 48, 58, 61, 62	15 items
Anger	3, 12, 17, 24, 31, 33, 39, 42, 47, 52, 53, 57	12 items
Vigor	7, 15, 19, 38, 51, 56, 60, 63	8 items
Fatigue	4, 11, 29, 40, 46, 49, 65	7 items
Confusion	8, 28, 37, 50, 54 *, 59, 64	7 items
Total		58 items

* score inverted.

**Table 3:** Athletes’ Self-Management Questionnaire (ASMQ).

Subscale	Item Number	
Physical conditioning	32, 26, 25, 5, 22	5 items
Training management	3, 2, 15, 29, 1, 11	6 items
Mental readiness	18, 24, 30, 9, 17, 10, 31, 34	8 items
Daily living management	20, 19, 8, 23, 21, 13, 16, 12	8 items
Interpersonal relationship	27, 35, 33	3 items
Personal routine	6, 28, 7, 14, 4	5 items
Total		35 items

**Table 4:** The Athletic Coping Skills Inventory-28 (ACSI-28).

Subscale	Item Number	
Coping with Adversity	5, 17, 21, 24	4 items
Peaking under Pressure	6, 18, 22, 28	4 items
Goal Setting/Mental Preparation	1, 8, 13, 20	4 items
Concentration	4, 11, 16, 25	4 items
Freedom from Worry	7 *, 12 *, 19 *, 23 *	4 items
Confidence and Achievement Motivation	2, 9, 14, 26	4 items
Coachability	3 *, 10 *, 15, 27	4 items
Personal Coping Resources	Total	28 items

* score inverted.

**Table 5:** Analysis of the moderating effect of tension on athletes’ self-management and sports coping skills.

Path	B	SE	*β*	*t*
Self-management → Athletic coping skills (b1)	0.370	0.044	0.576	8.377 ***
Tension → Athletic coping skills (b2)	−0.280	0.156	−0.120	−1.754
Interaction (b3)	−0.028	0.008	−0.242	−3.609 ***
R^2^	0.458			
ΔR^2^	0.059 ***			
*F*	33.764 ***			
	SD		CV	
Self-management	129.823		0.155	
Tension	13.879		0.401	
Athletic coping skills	65.968		0.196	

*** *p* < 0.001.

**Table 6:** The mediated effect wherein vigor affects sports coping skills through self-management.

Path	B	SE	*β*	*t*
Vigor → Self-management (a)	0.888	0.269	0.286	3.291 **
Vigor → Athletic coping skills (c)	0.833	0.164	0.417	5.074 ***
Self-management → Athletic coping skills (b)	0.354	0.045	0.550	7.843 ***
Vigor → Athletic coping skills (c’)	0.520	0.140	0.260	3.709 ***
	Effect	SE	LLCI	ULCI
Bootstrap	0.314	0.104	0.141	0.558
Z	3.013 **			
	SD		CV	
Vigor	15.089		0.430	
Self-management	129.823		0.155	
Athletic coping skills	65.968		0.196	

** *p* < 0.01, *** *p* < 0.001. LLCI, lower limit confidence interval; ULCI, upper limit confidence interval.

**Table 7:** The moderated mediation of vigor on sports coping skills through self-management.

Path	B	SE	*β*	*t*	LLCI	ULCI
1st step: dependent variable = Self-management						
Vigor (a)	0.887	0.269	0.286	3.291 **	0.353	1.420
R	0.286					
R^2^	0.082					
*F*	10.831 **					
2nd step: dependent variable = Athletic coping skills						
Vigor (a)	0.526	0.139	0.263	3.772 ***	0.250	0.802
Self-management (c)	0.636	0.110	0.496	7.223 ***	0.418	0.853
Tension (d)	−2.543	0.971	−0.178	2.664 *	0.620	4.466
Interaction (c × d)	−0.023	0.008	−0.197	−3.055 **	−0.038	−0.008
R	0.718					
R^2^	0.516					
*F*	31.670 ***					
	SD			CV		
Vigor	15.089			0.430		
Self-management	129.823			0.155		
Tension	13.879			0.401		

* *p* < 0.05, ** *p* < 0.01, *** *p* < 0.001. LLCI, lower limit confidence interval; ULCI, upper limit confidence interval.

**Table 8:** Direct effect and conditional indirect effect of vigor on sports coping skills.

Direct Effect of Vigor on Sports Coping Skills (Bootstrapping 95% CI)					
B	SE	*β*	*t*	LLCI	ULCI
0.526	0.139	0.263	3.771 ***	0.250	0.802
Depending on the level of tension, the conditional indirect effect of Vigor → Self-management → Sport coping skills					
Tension level	B	SE	*β*	LLCI	ULCI
M − 1 SD	0.395	0.120	0.198	0.189	0.656
Mean	0.283	0.092	0.142	0.129	0.499
M + 1 SD	0.171	0.085	0.085	0.038	0.377
		SD		CV	
Vigor		15.089		0.430	
Tension		13.879		0.401	

*** *p* < 0.001. LLCI, lower limit confidence interval; ULCI, upper limit confidence interval.

**Table 9:** Analysis of the moderated mediation.

Mediator	B	SE	*β*	LLCI	ULCI
Self-management	−0.020	0.009	−0.056	−0.040	−0.006
		SD		CV	
Self-management		129.823		0.155	

LLCI, lower limit confidence interval; ULCI, upper limit confidence interval.

## Data Availability

Not applicable.
